# The Potential Role of SCFAs in Modulating Cardiometabolic Risk by Interacting with Adiposity Parameters and Diet

**DOI:** 10.3390/nu16020266

**Published:** 2024-01-16

**Authors:** Joanna Ostrowska, Emilia Samborowska, Maciej Jaworski, Klaudia Toczyłowska, Dorota Szostak-Węgierek

**Affiliations:** 1 Department of Clinical Dietetics, Faculty of Health Sciences, Medical University of Warsaw, E Ciołka 27, 01-445 Warsaw, Poland; 2 Mass Spectrometry Laboratory, Institute of Biochemistry and Biophysics, Polish Academy of Sciences, Pawińskiego 5a, 02-106 Warsaw, Poland; 3 Department of Biochemistry, Radioimmunology and Experimental Medicine, The Children’s Memorial Health Institute, Al. Dzieci Polskich 20, 04-360 Warsaw, Poland

**Keywords:** SCFAs, body composition, VAT/SAT ratio, waist circumference, body shape index (ABSI), biochemical results, diet, dietary fiber, physical activity, total sleep time

## Abstract

The main objective of this cross-sectional study was to analyze the influence of lifestyle factors (diet, physical activity, sleep) that can affect the concentration of fecal short-chain fatty acids (SCFAs) and SCFAs’ potential role in modulating cardiometabolic disease risk by interacting with biochemical and body composition parameters. The study comprised 77 healthy, non-obese individuals aged 30–45 years who were assessed for the concentration of SCFAs in stool, diet, physical activity level, and sleep duration. Moreover, body composition measurement and patients’ biochemical parameters were included in the analysis. We have indicated a significant negative correlation between several SCFAs (especially acetic acid (AA), isobutyric acid (IBA), butyric acid (BA), propionic acid (PA), isovaleric acid (IVA) and valeric acid (VA)) with BMI, VAT/SAT ratio (visceral to subcutaneous fat ratio), and percentage of fat mass in a group of females enrolled in the study as well as with waist circumference (WC) in case of both sexes included in the study. Moreover, the results of our study acknowledged the importance of a diet in shaping the SCFA profile—we noticed significant negative associations between energy and fat intake and some SCFAs in males (IBA, IVA, VA, isocaproic acid (ICA)). Further, we indicated that a high intake of fiber (insoluble and soluble) in both males and females results in an elevated concentration of the vast majority of SCFAs and the amount of SCFAs in total. This effect was particularly noticeable in the case of the soluble fraction of fiber. These correlations reflect the fact that diet shapes the composition of the gut microbiota and SCFAs (main microbial metabolites) are synthesized from dietary fiber. In addition, we noticed that in a group of women, the concentration of AA, PA, and ICA as well as the total concentration of SCFAs showed a significant positive association with their sleep duration. We concluded that SCFAs can have a potential role in modulating cardiometabolic disease risk by interacting with adiposity parameters and diet. In addition, this potential direct link between diet and SCFAs may at least partly contribute to sleep improvement.

## 1. Introduction

Diet determines the gut microbiota structure, which has been identified as one of several potential pivotal factors illustrating dietary impact on health and disease [1,2]. Nonetheless, scrutinizing the link between health and microbiota in humans is demanding due to difficulties in maintaining proper control of environmental factors in individuals enrolling in the study. Several comprehensive studies have recently identified interactions between gut microbiota and diet that are associated with various cardiometabolic markers [2,3,4]; however, up to this point, only animal studies present some evidence of causality.

In this study, we scrutinized the association of lifestyle factors (diet, physical activity, sleep) with gut microbiota interaction focusing on the gut-derived microbial metabolites of SCFAs.

SCFAs are produced by fermenting non-digestible polysaccharides and play a regulatory role in improving gut health by enhancing intestinal barrier integrity [5], mucus production [6], and serotonin release [7]. Additionally, SCFAs play a vital role in metabolic regulation and can modulate cardiometabolic risk. Human intervention studies have shown that incorporating fiber-enriched diets in our daily routine can significantly improve the composition of gut microbiota, resulting in an increase in SCFAs production in stool or blood samples [8,9]. This evidence from human studies mainly seems to support the beneficial role of SCFAs in the regulation of blood glucose, blood lipids, and energy homeostasis. Nevertheless, robust conclusions about causality cannot be made. Moreover, several studies have indicated that elevated concentrations of SCFAs in stool samples are linked to adverse health outcomes such as gut microbiome dysbiosis, obesity, hypertension, and other risk factors for cardiometabolic diseases [10]. Further, SCFAs, considered as a source of energy, may be associated with overweight or obesity [2]. Consequently, the association of SCFAs with metabolic disorders and obesity is ambiguous. Furthermore, our study delved beyond basic anthropometric and body composition measures to investigate more advanced indicators directly linked to cardiometabolic risk, such as the visceral-to-subcutaneous-fat ratio (VAT/SAT ratio) and a body shape index (ABSI). ABSI adjusts waist circumference (WC) for BMI and is nearly independent of BMI [11], while both ABSI and the VAT/SAT ratio have been found to detect abdominal adiposity [12,13]. As far as we know, our study is among the few to explore the relationship between these indicators and SCFAs.

Therefore, in the present study, we aimed to (1) analyze the stool concentration of SCFAs in healthy non-obese men and women and (2) analyze the association of lifestyle factors (diet, physical activity, sleep), with the concentrations of fecal SCFAs and the association of SCFAs with cardiometabolic disease risk, including adiposity parameters such as the VAT/SAT ratio, WC, ABSI and selected biochemical parameters.

## 2. Materials and Methods

### 2.1. Subjects and Data Collection

The cross-sectional study was carried out at the Medical University of Warsaw, in 77 healthy, non-obese adults (31 men and 46 women), who voluntarily took part in the research based on the advertisement. The inclusion criteria were age 30–45 years, no diagnosed chronic diseases, and BMI in the range 18.5 kg/m^2^–29.9 kg/m^2^. Exclusion criteria included (1) pharmacological treatment, (2) supplement intake besides vitamin D intake, (3) past abdominal surgery, (4) elimination diets, (5) excessive alcohol consumption (more than 20 g of pure alcohol per day for women and 30 g of pure alcohol per day for men), (6) use of probiotics in the three months prior to joining the study, and (7) use of antibiotics and chemotherapy drugs in the three months before joining the study, as well as any contraindications that apply to body composition analysis (confirmed epilepsy, implanted cardiac pacemaker, defibrillator, and metal endoprosthesis). Weight, height and waist circumference were measured using a measurement station, Seca 799 column scales and a steel measuring tape, respectively. Based on the obtained data, the following formula was used to calculate a body shape index (ABSI): (WC in meters)/[(BMI^2/3) × (height in meters)^1/2].

### 2.2. Bioelectrical Impedance Analysis

We used the bioelectrical impedance method to analyze the body composition of participants. The Bioscan 920-2 device from Maltron Int, UK (Rayleigh, Essex, UK), was used according to the manufacturer’s instructions. The participants were required to comply with the guidelines issued by the European Society for Clinical Nutrition and Metabolism (ESPEN) [14]. On the day of the examination, the participants were instructed to fast for at least 2 to 3 h before the test, avoid physical activity for 12 h before the test, abstain from alcohol, caffeinated beverages, or fizzy drinks for at least 24 h before the test, and empty their bladder 30 min before the test.

### 2.3. Biochemical Tests

Fasting serum concentration of insulin, glucose, total cholesterol, triglycerides, LDL-cholesterol, HDL-cholesterol, and high-sensitivity C-reactive protein were measured in all participants. Homeostatic model assessment of insulin resistance (HOMA-IR) index was calculated from the fasting glucose and insulin concentration. Blood samples were obtained from participants in the morning after a 12-h fast without them taking any medications.

### 2.4. Physical Activity (PA) Measured by Accelerometer

Participants wore tri-axial accelerometers (wGT3X-BT ActiGraph LLC, Pensacola, FL, USA) on a waist belt aligned with the right anterior axillary line for 7 days. The epoch length was 30 s, and the minimum wear time was 10 h/day for 4 days, including a weekend day. The physical activity time was determined based on count thresholds for moderate (MPA) or vigorous intensity physical activity (VPA), separately or combined (MVPA), using Freedson’s cut-offs [15]. Activity time at defined intensities (MPA, VPA, or MVPA) was calculated by adding up minutes in a day (using Actilife 5.5 software).

### 2.5. Sleep Duration Measured Using an Accelerometer

Sleep duration was monitored for 7 days using an electronic device (wGT3X-BT ActiGraph LLC, Pensacola, FL, USA). The Cole–Kripke algorithm [16] was used to calculate sleep and wake periods. Data from participants with less than 4 nights of data were excluded from analyses. Data were separated into sleep and wake periods by visual inspection of the actigraphy tracings (using Actilife 5.5 software).

### 2.6. Nutritional Value of Daily Food Consumption

To assess the participants’ diet, data were gathered from a three-day dietary record, spanning two weekdays and one weekend day. During this time, participants used standard measurement units like glass, cup, bowl, spoon, package sizes, and weights to report the quantity of food consumed. Before recording their dietary intake, the participants were instructed by a dietitian on how to estimate food intake using household utensils and how to properly record their dietary intake. To ensure the accuracy of the records, participants were encouraged to attach photos of the food they described. After completing the 3-day dietary records, the dietitian cross-checked the information during an in-person meeting and clarified any doubts if needed, including the adjustment of obtained information using the album of photographs of food products and dishes elaborated by the National Food and Nutritional Institute in Warsaw. The calculation of energy, macronutrient content, and dietary fiber was performed using Dieta 6.0 nutritional software, developed by the National Food and Nutrition Institute in Warsaw, Poland. The data related to the fiber content, including both soluble and insoluble fibers, were obtained from the annually updated Fineli database, maintained by the Finnish Institute for Health and Welfare (https://fineli.fi/fineli/en/index).

### 2.7. SCFAs

#### 2.7.1. Chemicals

All short-chain fatty acids standards ((acetic acid-AA (C2), propionic acid- PA (C3), butyric acid-BA (C4), isobutyric acid-IBA (C4), valeric acid- VA (C5), isovaleric acid-IVA (C5), caproic acid—CA (C6), isocaproic acid ICA (C6)) and short-chain fatty acid isotope-labeled standards (acetic acid-13C2, propionic acid-D6, butyric acid-13C2, isobutyric acid-D7, valeric acid-D9), pyridine anhydrous, 2-nitro phenylhydrazine (3NPH·HCl), N-(3-dimethylaminopropyl)-N′-ethylcarbodiimide (EDC·HCl)) were acquired from Sigma-Aldrich (St. Louis, MO, USA). All SCFAs stock solutions were prepared in 50% acetonitrile and stored at −20 °C. LC-MS grade acetonitrile, HPLC-grade acetonitrile, HPLC-grade methanol, and formic acid were obtained from J.T. Baker. Ultra-pure water (Mili-Q water) was produced using a water purification system (Mili-Q, Millipore, Milford, MA, USA).

#### 2.7.2. Sample Preparation

A sample of stools was weighed and homogenized (100 mg sample per 400 µL of 0.9% NaCl), and the sample was centrifuged for 5 min at 5000 rpm. The supernatant was transferred to a new laboratory tube. Sample preparation was as follows: 40 µL of sample and 80 µL methanol (containing internal standards) were mixed for 1 min. Next, 20 µL of 3NPH solution and 20 µL of EDC-pyridine solution were added and the mixture was incubated at room temperature for 30 min. The solution was diluted to 1 mL with 15% aqueous acetonitrile and centrifuged, and then 10 µL of the mixture was injected into the apparatus.

#### 2.7.3. Analyzes

SCFA quantification was performed using a Waters Acquity ultra-performance liquid chromatograph coupled with a Waters TQ-S triple-quadrupole mass spectrometer. LC/MS/MS analysis was performed in negative electrospray ionization (ESI)-multiple-reaction monitoring (MRM) mode. The SCFAs were separated using a Waters BEH C18 column (1.7 µm, 2.1 mm × 50 mm) and a Waters BEH C18 guard column (1.7 µm, 2.1 mm × 5 mm). A measure of 1 mL of formic acid in 1 L water was used as mobile phase A, and 1 mL of formic acid in acetonitrile was used as mobile phase B. The flow rate of the mobile phase was set at 0.6 mL/min.

The limits of quantification (LOQ) were 10 μM for acetic acid, propionic acid, and butyric acid and 0.1 μM for isovaleric acid, valeric acid, and caproic acid.

Chromatograms of SCFAs and their internal standards (IS) of representative fecal samples are shown in Supplementary Figures S1 and S2. 

### 2.8. Statistical Analyzes

The Shapiro–Wilk test was used for testing departure of analyzed variables from the Gaussian distribution. Since variables showed significant departure from parametric distribution, non-parametric statistics were used. Presented are the median (and inter-quartile range), Spearman correlation r, and *p*-value from the Mann–Whitney test. Statistica v. 10 (StatSoft Inc., Tulsa, OK, USA) was used for statistical calculations. A *p*-value less than 0.05 was considered significant.

## 3. Results

### 3.1. Anthropometric and Biochemical Characteristics

A total of 77 healthy non-obese participants (31 men and 46 women) were enrolled. The mean age, height, weight, and BMI were as follows: 37 years, 72 kg, 173 cm, and 24 kg/m^2^, respectively. There were no significant differences in age between males and females (*p* > 0.05), whereas all body composition and anthropometric indicators were significantly different (*p* < 0.05). No sex-specific differences in HOMA-IR, TC, LDL-C, or CRP values were observed. However, significantly higher TG values and lower HDL-C levels were found in males compared to females (119 vs. 78 mg/dL, <0.001 and 53 vs. 67 mg/dL, *p* < 0.001 respectively). When it comes to lifestyle factors, the levels of physical activity (PA) (moderate PA, as well as vigorous PA, jointly referred to as MVPA) were observed as significantly higher in males in comparison to females (90 vs. 57 min/day, *p* < 0.05). The same sex-specific differences were observed in energy, protein, fats, and fiber intake, whereas at the same time, no differences in sleep duration or fecal SCFAs percentage were observed.

The descriptive characteristics of the study population are shown in Table 1.

### 3.2. Correlation of Lifestyle Factors and SCFAs

In the correlation analysis conducted in a group of women, the total concentration of SCFAs showed a significant positive association (*p* < 0.05) with total sleep time and total fiber consumption—both insoluble and soluble—while in the case of the VAT/SAT ratio, we noticed a negative correlation with the total amount of SCFAs. In addition, a positive correlation was observed between some SCFAs and the percentage of FFM and TBW—mainly isobutyric and isovaleric acids—while both of the above-mentioned SCFAs were significantly negatively correlated (*p* < 0.005) with the percentage of fat mass. In the described correlation analysis, fecal SCFAs showed no association with physical activity and laboratory outcomes (Table 2) (Supplementary Figure S3).

After discovering an interesting link between total sleep time and certain SCFAs, we decided to delve deeper into the correlation between TST and other analyzed anthropometric, biochemical, and lifestyle parameters. We found that in a group of women enrolled in the study, TST is significantly negatively correlated with carbohydrate consumption (r = −0.32, *p*-value < 0.05).

In a group of males, we found no correlations between fecal SCFAs and body composition parameters, except for a few anthropometric indicators such as waist circumference (significant negative association with propionic and butyric acid). However, there was a positive association (*p* < 0.05) between several SCFAs and laboratory outcomes such as TG, CRP, and fasting blood glucose. In addition, in the group of men, as in the group of women, we observed a significant influence of factors related to diet on the concentration of SCFAs. The most noticeable correlations were indicated in the case of fiber intake (significant positive association) and energy and fat intake (significant negative association). In terms of fiber intake, we noticed an elevated concentration of almost all analyzed SCFAs. This observation pertains to the fiber in total, including its division into insoluble and soluble fractions. However, butyric acid displayed a higher degree of statistical significance in relation to soluble fiber (Table 3) (Supplementary Figure S4).

### 3.3. Fecal SCFA Profile: Percentage of SCFAs Depending on Various Fiber Intake

Considering that the diet factors have a strong correlation with the concentration of fecal SCFAs, we divided study participants into two groups according to fiber intake, to check whether the percentage profile of SCFAs had changed. These two groups were selected for both women and men enrolled in the study. There were statistically significant differences in the percentage of valeric acid between the female groups divided based on fiber intake. In the group of men enrolled in the study, we noticed that these individuals who reported a higher fiber intake had statistically significantly elevated percentages of butyric acid and valeric acid (Table 4 and Table 5). The other analyzed SCFAs did not differ significantly depending on fiber intake in both genders.

## 4. Discussion

Acetic, propionic, and butyric acids are the most abundant SCFAs present in the colon. Typically, the molar ratio of these SCFAs remains consistent at 60:20:20, respectively, under normal physiological conditions [17]. Our research found that proportions of acetic, propionic, and butyric acid and other SCFAs were similar both in the group of women (62:16:18:3:1, respectively) and in the group of men (60:16:18:5:1, respectively), with no sex-specific statistically significant differences.

SCFAs play a vital metabolic role—scientific research has revealed that they aid in the activation of G protein-coupled receptors, including GPR41 and GPR43, and help in preventing the accumulation of fat in adipose tissue. Additionally, SCFAs improve the metabolism of unincorporated lipids and glucose in other tissues, resulting in improved insulin sensitivity [18,19]. Moreover, SCFAs facilitate the release of satiety hormones such as glucagon-like peptide-1 and peptide YY, further contributing to their beneficial effects [20,21]. Evidence suggests that fecal SCFAs are negatively correlated with adiposity parameters, including VAT and WC [22,23,24]. Furthermore, it was indicated that dietary supplementation with acetate, propionate, butyrate, or a combination of these can effectively hinder weight gain caused by a high-fat diet [25]. In our study, we also indicate a significant negative correlation between acetic, butyric, isobutyric, propionic, isovaleric and valeric acid with BMI, VAT/SAT ratio, and percentage of fat in a group of females. In addition, propionic and butyric acids have a significant negative association with WC in males. Besides this, we also showed the same significant negative associations for valeric acid with fat and WC in females, at the same time having a significant positive association with the percentage of TBW and FFM. All our findings support the above-mentioned thesis that SCFAs (in our study especially acetic, butyric, and valeric acid) may protect healthy individuals against overweight and obesity. However, some other authors came to different conclusions, and the positive association between elevated fecal SCFAs and cardiometabolic disease risk factors, obesity, or gut microbiome dysbiosis has been demonstrated in several human studies [26,27,28,29]. These findings may be explained by low SCFA absorption in epithelial cells in the gut and the occurrence of chronic inflammation associated with overweight and obesity. These mechanisms warrant further investigation, but it is worth mentioning that the concentration of SCFAs in feces is determined by intestinal bacteria’s production of these compounds, their absorption, and expenditure in the gastrointestinal tract [29]. Moreover, SCFAs as an energy source may be linked to the development of overweight or obesity [27]. Moreover, some studies have shown that the acetic acid produced by the intestinal microbiota is associated with de novo lipogenesis and stimulation of hepatic cholesterogenesis, which may lead to insulin resistance and an increased concentration of serum fasting blood glucose [30]. This dependence was partially confirmed in our research—we indicated the significant relationship between acetic acid and fasting blood glucose in a group of men. However, we did not observe a statistically significant relationship between acetic acid and the composition of the host’s lipids. Similar results were obtained in a study by Granado-Serrano et al. [31], where no significant difference in the concentration of acetic acid in the serum between individuals with hypercholesterolemia and those with normocholesterolemia was found. Besides the above-mentioned association, significant positive correlations were also notable in the case of other laboratory test results—such as valeric acid and CRP, as well as caproic acid and TG. All described correlations we observed only in the group of men, which may be explained by the higher prevalence of the occurrence of cardiometabolic risk factors in men [32,33,34], which was also confirmed in our study (e.g., lower concentration of HDL and higher concentration of TG in comparison to women) (Table 1).

Evidence suggests that SCFAs may affect sleep via gut–brain communication due to their ability to cross the blood–brain barrier [35]. There is growing evidence indicating that signals produced by the intestinal microbiota can promote sleep [35,36,37]. These findings are consistent with outcomes from our study—we noted that acetic, propionic, and isocaproic acids as well as total SCFAs in stool were positively associated with longer total sleep time in females. After discovering an interesting link between total sleep time and certain SCFAs, we decided to delve deeper into the correlation between TST and other analyzed anthropometric, biochemical, and lifestyle parameters in men and women enrolled in the study. No significant relationship was found between fiber intake and sleep duration. Therefore, based on this study, we cannot demonstrate a clear link between fiber intake, SCFAs, and sleep. Further research may be necessary to gain a deeper understanding of this topic. Nevertheless, we found that, in a group of women, TST is significantly negatively correlated with carbohydrate consumption (r = −0.32, *p*-value < 0.05). The current scientific results highlight the effect of diet, including carbohydrates, on sleep. Serval studies have investigated the relationship between carbohydrate consumption and sleep, although the findings are not entirely consistent and may depend on various factors, such as the type of carbohydrate, timing of intake, and individual response [38]. Nevertheless, there are studies—including our own—that suggest a potential negative effect of high carbohydrate intake on sleep duration [39,40].

The connection between physical activity and microbiota composition can be explained through several mechanisms. These include immune modulation, antioxidant activity, gastrointestinal permeability, and the production of metabolites such as SCFAs [41]. However, in our study, we did not demonstrate any associations between physical activity and SCFAs. In addition, it is worth mentioning that associations between physical activity and SCFAs have not been extensively studied, the results of existing publications are inconsistent [41,42,43,44,45,46], and the mechanism by which physical activity modulates SCFAs and vice versa remains speculative [42].

Changes in diet can lead to positive effects on overall health through SCFAs increased production [47]. Studies have shown that augmenting the intake of dietary fiber leads to a rise in the concentration of SCFAs in the gut [48,49]. Conversely, a diet that is rich in fat but low in fiber has been found to decrease SCFA levels [50]. Wan et al. [51] conducted a controlled feeding trial aimed at comparing three dietary patterns with varying proportions of fat and carbohydrates. The study included 217 healthy young adults who were monitored for 6 months. The researchers found that the high-fat diet (40% energy from fat) had negative effects on the fecal bacterial metabolites, gut microbiota, and markers of inflammation. Conversely, a lower-fat diet (20% energy from fat) was associated with a more advantageous impact on these biomarkers. The results of our study also underline the importance of diet—we noticed significant negative associations between energy and fat intake and some SCFAs (isobutyric, isovaleric, valeric, and isocaproic acids). This finding may support the thesis that a high-fat and high-energy diet lowers levels of SCFAs in the intestine and that the introduction of a low-energy diet may increase the quantity and richness of the microorganisms and SCFAs [48,51,52]. In terms of fiber consumption, the analysis has shown that a rise in fiber intake leads to elevated concentration of acetic, propionic, and butyric acid as well as SCFAs in total, for both genders. Additionally, in men, caproic and valeric acid were also noted as being elevated. Most of these significant associations have been verified for a specific type of fiber—namely, insoluble and soluble. Among these two, the soluble fraction showed greater statistical significance, particularly among females. The above-mentioned results are consistent with the literature. Although both types of fiber are known to promote the production of SCFAs, soluble fiber is considered more advantageous due to its extensive fermentation by gut bacteria [52,53]. All these correlations reflect the fact that SCFAs are synthesized from dietary fiber and a diet comprising a variety of fiber-rich foods leads to an increase in the synthesis of SCFAs [48,52]. We also showed this correlation in the percentage distribution analysis of SCFAs—our results confirmed the significantly elevated percentage of butyric acid and valeric acid in the groups of individuals with high fiber intake (≥25 g) (Table 4 and Table 5).

It is important to consider certain limitations of the current investigation while analyzing the data. The primary limitation of this research was the small size of the participant sample. To draw more objective conclusions, a study that includes a large group of both men and women would be required. Additionally, supplementing our study with an analysis of SCFAs in the circulation and the microbiome profile of each participant undoubtedly enriches our findings.

## 5. Conclusions

Our findings support the thesis that SCFAs may protect healthy individuals against overweight and obesity and consequently have a potential role in modulating cardiometabolic disease risk by interacting with adiposity parameters and diet (mainly dietary fiber and its soluble fraction). However, we obtained inconsistent findings regarding biochemical outcomes in a group of men—a significant positive correlation between some fecal SCFAs and fasting blood glucose, CRP, and TG. These contradictory effects require further investigation.

Analyzing lifestyle factors, we did not identify any associations between physical activity and SCFAs. At the same time, we concluded that a potential direct link between diet and SCFAs—indicated in our study—may at least partly contribute to sleep mechanisms and sleep improvement, which was shown in the case of women enrolled in the study (however, findings in existing publications are scarce and inconsistent).

## Figures and Tables

**Table 1:** Characteristics of study participants.

	Total *n* = 77		Females *n* = 46		Males *n* = 31		
	Mean	SD	Mean	SD	Mean	SD	*p*-Value
Basic parameters							
Age (years)	36.75	4.69	36.26	4.46	37.48	5.00	0.354
Body weight (kg)	72.07	14.42	62.99	8.25	85.55	10.51	<0.001
Height (cm)	172.75	9.69	166.72	6.51	181.69	6.01	<0.001
BMI (kg/m^2^)	23.96	3.12	22.64	2.52	25.91	2.93	<0.001
WC (cm)	83.86	11.61	77.60	8.16	93.16	9.60	<0.001
ABSI	0.077	0.005	0.075	0.004	0.079	0.004	<0.001
Body composition parameters							
VAT (cm^2^)	118.21	82.94	84.02	51.25	168.94	95.05	<0.001
SAT (cm^2^)	97.73	35.41	88.59	32.90	111.29	35.14	0.006
VAT/SAT	1.15	0.58	0.93	0.31	1.49	0.72	<0.001
FFM (kg)	51.98	10.40	44.39	3.71	63.25	5.80	<0.001
FFM (%)	72.27	5.62	71.05	5.63	74.10	5.18	0.022
FM (kg)	20.34	6.52	18.69	5.65	22.78	7.03	0.011
FM (%)	27.74	5.54	29.04	5.46	25.81	5.17	0.016
TBW (Lt)	36.98	7.86	31.24	3.00	45.50	4.19	<0.001
TBW (%)	51.28	3.62	49.93	3.14	53.28	3.40	<0.001
Biochemical parameters							
TC (mg/dL)	199.32	29.80	199.19	26.60	199.53	34.46	0.775
HDL-C (mg/dL)	61.46	14.55	67.22	14.57	52.90	9.53	<0.001
LDL-C (mg/dL)	120.35	23.88	116.37	21.42	126.26	26.38	0.162
TG (mg/dL)	94.87	47.72	78.35	26.63	119.38	60.55	<0.001
CRP (mg/L)	1.54	2.85	1.20	1.21	2.05	4.23	0.270
Fasting blood glucose (mg/dL)	97.55	7.33	96.57	5.79	99.00	9.06	0.192
Fasting insulin (μU/mL)	8.18	4.64	7.12	2.88	9.77	6.14	0.153
HOMA-IR	2.00	1.24	1.71	0.74	2.44	1.65	0.111
Physical activity and sleep parameters							
MPA (min/day)	61.69	31.34	52.53	16.89	75.29	41.71	0.034
VPA (min/day)	9.00	15.32	5.06	7.55	14.85	21.21	0.039
MVPA (min/day)	70.48	43.72	57.39	19.51	89.92	60.15	0.037
TST (h/night)	7.27	1.27	7.45	1.41	7.01	0.98	0.136
Short-chain fatty acids in stool							
C 2:0 (AA) (%)	60.80	6.02	61.62	5.83	59.57	6.19	0.122
C 3:0 (PA) (%)	15.67	3.49	15.62	2.97	15.74	4.20	0.640
C 4:0 i (IBA) (%)	2.49	1.31	2.61	1.33	2.30	1.27	0.383
C 4:0 n (BA) (%)	14.81	6.08	13.99	5.92	16.02	6.20	0.161
C 5:0 i (IVA) (%)	2.25	1.35	2.39	1.42	2.06	1.25	0.345
C 5:0 n (VA) (%)	2.73	0.94	2.65	1.02	2.85	0.82	0.406
C 6:0 i (ICA) (%)	0.04	0.04	0.04	0.03	0.04	0.04	0.934
C 6:0 n (CA) (%)	1.22	1.36	1.08	0.97	1.42	1.79	0.771
Diet parameters							
Energy (kcal/d)	2040.59	448.92	1803.27	263.01	2413.53	428.68	<0.001
Protein (g/d)	85.35	23.90	73.37	16.53	104.18	21.57	<0.001
Fats (g/d)	77.83	21.02	70.50	14.85	89.34	24.21	<0.001
Carbohydrates (g/d)	242.06	63.76	220.98	39.35	275.20	79.75	0.001
Total fiber (g/d)	25.12	9.09	23.28	8.84	28.02	8.87	0.027
Insoluble fiber (g/d)	16.35	6.2	14.9	6.2	18.4	5.6	0.01
Soluble fiber (g/d)	8.71	4.6	8.7	3.7	10.3	4.7	0.15

Abbreviations: BMI—body mass index, WC—waist circumference, ABSI—body shape index, FFM—fat free mass, FM—fat mass, VAT—visceral adipose tissue, SAT—subcutaneous adipose tissue, TBW—total body water, HOMA-IR-homeostatic model assessment insulin resistance, TC—total cholesterol, TG—triglycerides, HDL-C—high density lipoprotein cholesterol, LDL-C—low density lipoprotein cholesterol, CRP—c-reactive protein, MPA—moderate physical activity, VPA—vigorous physical activity, MVPA—moderate and vigorous physical activity, TST—total sleep time, C 2:0—acetic acid (AA), C 3:0—propionic acid (PA), C 4:0 i—isobutyric acid (IBA), C 4:0 n butyric acid (BA), C 5:0 i—isovaleric acid (IVA), C 5:0 n—valeric acid (VA), C 6:0 i—isocaproic acid (ICA), C 6:0 n—caproic acid (CA).

**Table 2:** Correlations of anthropometric, biochemical, and lifestyle parameters and fecal SCFAs—females (*n* = 46).

	C 2:0 (AA)	C 3:0 (PA)	C 4:0 i (IBA)	C 4:0 n (BA)	C 5:0 i (IVA)	C 5:0 n (VA)	C 6:0 i (ICA)	C 6:0 n (CA)	Total
Age (yr)	−0.04	−0.06	0.16	−0.01	0.15	0.09	0.07	0.16	−0.03
BMI (kg/m^2^)	−0.14	−0.10	−0.34 *	−0.15	−0.33 *	−0.29 *	0.04	−0.18	−0.15
WC (cm)	−0.27	−0.17	−0.26	−0.23	−0.22	−0.31 *	−0.06	−0.12	−0.27
ABSI	−0.23	−0.09	−0.02	−0.11	0.03	−0.14	−0.09	0.04	−0.20
VAT/SAT	−0.36 *	−0.30 *	−0.07	−0.39 **	0.01	−0.18	−0.11	0.00	−0.36 *
FFM (%)	0.11	0.07	0.39 **	0.14	0.39 **	0.32 *	0.00	0.17	0.14
FM (%)	−0.11	−0.07	−0.39 **	−0.13	−0.39 **	−0.32 *	0.01	−0.17	−0.13
TBW (%)	0.09	0.08	0.33 *	0.11	0.33 *	0.22	0.02	0.04	0.11
TC (mg/dL)	−0.21	−0.15	−0.18	−0.07	−0.15	−0.16	−0.29	−0.05	−0.18
HDL-C (mg/dL)	0.02	−0.02	−0.14	0.19	−0.07	−0.03	−0.16	0.17	0.05
LDL-C (mg/dL)	−0.26	−0.18	−0.12	−0.24	−0.12	−0.16	−0.23	−0.14	−0.25
TG (mg/dL)	0.01	0.03	0.00	0.03	−0.03	−0.05	0.08	−0.13	0.00
CRP (mg/L)	−0.15	−0.08	−0.02	−0.02	−0.02	0.07	0.06	0.18	−0.14
Fasting blood glucose (mg/dL)	−0.04	−0.09	−0.07	−0.10	−0.01	0.00	0.03	0.11	−0.05
Fasting insulin (μU/mL)	−0.13	0.02	0.02	−0.24	0.03	0.02	0.03	−0.03	−0.13
HOMA-IR	−0.13	0.01	0.02	−0.25	0.03	0.02	0.02	−0.02	−0.14
MVPA (min/d)	−0.26	−0.10	−0.07	−0.10	−0.07	−0.24	−0.07	−0.12	−0.21
TST (h/night)	0.34 *	0.33 *	−0.09	0.23	−0.12	0.12	0.32 *	−0.01	0.33 *
Energy (kcal/d)	0.04	0.04	−0.21	0.19	−0.14	−0.03	−0.05	0.08	0.08
Carbohydrates (g/d)	0.16	0.12	−0.28	0.25	−0.29	−0.07	−0.07	0.05	0.25
Protein (g/d)	0.08	0.04	0.03	0.16	0.07	0.10	−0.07	0.12	0.12
Fats (g/d)	0.14	0.12	0.07	0.25	0.12	0.20	0.11	0.13	0.21
Total fiber (g/d)	0.36 *	0.30 *	−0.14	0.45 **	−0.15	0.11	0.10	0.03	0.38 *
Insoluble fiber (g/d)	0.30 *	0.27	−0.15	0.44 **	−0.12	0.11	0.05	0.11	0.34 *
Soluble fiber (g/d)	0.45 **	0.39 **	−0.11	0.48 **	−0.17	0.21	0.25	0.11	0.46 **

The Spearman correlation coefficient, *r*_s_ * *p* < 0.05, ** *p* < 0.01. Abbreviations: BMI—body mass index, WC—waist circumference, ABSI—body shape index, FFM—fat free mass, FM—fat mass, VAT—visceral adipose tissue, SAT—subcutaneous adipose tissue, TBW—total body water, HOMA-IR—homeostatic model assessment insulin resistance, TC—total cholesterol, TG—triglycerides, HDL-C—high density lipoprotein cholesterol, LDL-C—low density lipoprotein cholesterol, CRP—c-reactive protein, MVPA—moderate and vigorous physical activity, TST—total sleep time, C 2:0—acetic acid (AA), C 3:0—propionic acid (PA), C 4:0 i—isobutyric acid (IBA), C 4:0 n butyric acid (BA), C 5:0 i—isovaleric acid (IVA), C 5:0 n—valeric acid (VA), C 6:0 i—isocaproic acid (ICA), C 6:0 n—caproic acid (CA).

**Table 3:** Correlations of anthropometric, biochemical, and lifestyle parameters and fecal SCFAs—males (*n* = 31).

	C 2:0 (AA)	C 3:0 (PA)	C 4:0 i (IBA)	C 4:0 n (BA)	C 5:0 i (IVA)	C 5:0 n (VA)	C 6:0 i (ICA)	C 6:0 n (CA)	Total
Age (yr)	−0.15	−0.11	0.06	−0.05	0.04	0.07	0.05	0.21	−0.14
BMI (kg/m^2^)	−0.10	−0.23	−0.12	−0.21	−0.16	−0.11	−0.03	0.21	−0.17
WC (cm)	−0.19	−0.36 *	−0.07	−0.38 *	−0.10	−0.15	−0.12	0.07	−0.32
ABSI	−0.15	−0.27	0.12	−0.32	0.13	0.02	−0.13	0.02	−0.26
VAT/SAT	−0.08	−0.17	0.22	−0.22	0.19	0.11	−0.10	0.10	−0.15
FFM (%)	0.06	0.17	0.09	0.16	0.08	0.07	−0.07	−0.12	0.13
FAT (%)	−0.10	−0.18	−0.09	−0.23	−0.10	−0.12	0.05	0.06	−0.18
TBW (%)	−0.06	0.04	0.24	0.08	0.23	0.07	0.04	0.04	0.04
TC (mg/dL)	0.11	0.11	−0.14	0.15	−0.07	−0.01	−0.15	0.09	0.15
HDL-C (mg/dL)	−0.18	−0.06	−0.20	−0.16	−0.15	−0.30	−0.25	−0.19	−0.16
LDL-C (mg/dL)	0.19	0.16	−0.09	0.31	0.01	0.09	−0.05	0.08	0.24
TG (mg/dL)	0.01	−0.06	0.18	0.06	0.18	0.22	0.05	0.38 *	0.06
CRP (mg/L)	0.08	0.18	0.28	0.25	0.33	0.37 *	0.27	0.13	0.17
Fasting blood glucose (mg/dL)	0.36 *	0.35	0.01	0.19	−0.05	0.04	−0.09	−0.35	0.30
Fasting insulin (μU/mL)	0.08	0.03	−0.09	−0.14	−0.17	−0.08	−0.04	−0.20	−0.03
HOMA-IR	0.12	0.06	−0.09	−0.10	−0.17	−0.06	−0.03	−0.21	0.01
MVPA (min/d)	0.33	0.27	−0.02	0.18	−0.10	0.12	0.08	0.17	0.28
TST (h/night)	−0.35	−0.29	0.03	−0.37	0.09	−0.19	−0.01	0.13	−0.31
Energy (kcal/d)	0.11	0.11	−0.37 *	0.09	−0.47 *	−0.09	−0.09	0.01	0.06
Protein (g/d)	0.26	0.23	−0.32	0.19	−0.37	−0.07	−0.02	0.02	0.24
Carbohydrates (g/d)	0.14	0.20	−0.09	0.28	−0.19	0.26	0.32	0.35	0.14
Fats (g/d)	0.11	−0.16	−0.55 **	−0.05	−0.58 **	−0.49 **	−0.56 **	−0.24	−0.02
Total fiber (g/d)	0.61 ***	0.39 *	−0.16	0.64 ***	−0.16	0.48 **	0.12	0.39 *	0.62 ***
Insoluble fiber (g/d)	0.46 *	0.20	0.09	0.47 *	0.08	0.43 *	0.08	0.31	0.45 *
Soluble fiber (g/d)	0.44 *	0.21	−0.21	0.48 **	−0.21	0.08	0.06	0.09	0.44 *

The Spearman correlation coefficient, *r*_s_ * *p* < 0.05, ** *p* < 0.01, and *** *p* < 0.001. Abbreviations: BMI—body mass index, WC—waist circumference, ABSI—body shape index, FFM—fat free mass, FM—fat mass, VAT—visceral adipose tissue, SAT—subcutaneous adipose tissue, TBW—total body water, HOMA-IR-homeostatic model assessment insulin resistance, TC—total cholesterol, TG—triglycerides, HDL-C—high density lipoprotein cholesterol, LDL-C—low density lipoprotein cholesterol, CRP—c-reactive protein, MVPA—moderate and vigorous physical activity, TST—total sleep time, C 2:0—acetic acid (AA), C 3:0—propionic acid (PA), C 4:0 i—isobutyric acid (IBA), C 4:0 n butyric acid (BA), C 5:0 i—isovaleric acid (IVA), C 5:0 n—valeric acid (VA), C 6:0 i—isocaproic acid (ICA), C 6:0 n—caproic acid (CA).

**Table 4:** Differences in fecal SCFAs percentage depending on various fiber intake—females.

	Fiber Intake ≥ 25 g (*n* = 17)		Fiber Intake < 25 g (*n* = 27)		
SCFA (%)	Median	IQR	Median	IQR	*p*-Value *
C 2:0 (AA) (%)	60.31	8.46	60.35	6.11	0.376
C 3:0 (PA) (%)	15.75	3.89	15.06	4.09	0.599
C 4:0 i (IBA) (%)	2.02	2.54	2.87	1.59	0.157
C 4:0 n (BA) (%)	17.50	8.02	12.54	8.66	0.070
C 5:0 i (IVA) (%)	1.66	2.37	2.66	1.82	0.179
C 5:0 n (VA) (%)	2.17	0.93	2.81	0.71	0.042
C 6:0 i (ICA) (%)	0.03	0.03	0.03	0.03	0.703
C 6:0 n (CA) (%)	0.54	1.11	1.11	1.44	0.390

* Mann–Whitney test. Abbreviations: C 2:0—acetic acid (AA), C 3:0—propionic acid (PA), C 4:0 i—isobutyric acid (IBA), C 4:0 n butyric acid (BA), C 5:0 i—isovaleric acid (IVA), C 5:0 n—valeric acid (VA), C 6:0 i—isocaproic acid (ICA), C 6:0 n—caproic acid (CA).

**Table 5:** Differences in fecal SCFAs percentage divided depending on various fiber intake—males.

	Fiber Intake ≥ 25 g (*n* = 17)		Fiber Intake < 25 g (*n* = 11)		
SCFA (%)	Median	IQR	Median	IQR	*p*-Value *
C 2:0 (AA) (%)	57.98	7.42	59.25	11.88	0.175
C 3:0 (PA) (%)	13.71	4.94	14.26	8.41	0.487
C 4:0 i (IBA) (%)	1.49	0.85	3.46	1.51	<0.001
C 4:0 n (BA) (%)	19.22	5.09	11.97	9.39	<0.001
C 5:0 i (IVA) (%)	1.23	1.14	3.16	1.62	0.002
C 5:0 n (VA) (%)	2.71	1.08	2.79	1.42	0.430
C 6:0 i (ICA) (%)	0.03	0.03	0.05	0.07	0.264
C 6:0 n (CA) (%)	1.60	1.89	0.97	1.18	0.329

* Mann–Whitney test. Abbreviations: C 2:0—acetic acid (AA), C 3:0—propionic acid (PA), C 4:0 i—isobutyric acid (IBA), C 4:0 n butyric acid (BA), C 5:0 i—isovaleric acid (IVA), C 5:0 n—valeric acid (VA), C 6:0 i—isocaproic acid (ICA), C 6:0 n—caproic acid (CA).

## Data Availability

Data are contained within the article.

## Supplementary Materials

The following supporting information can be downloaded at: https://www.mdpi.com/article/10.3390/nu16020266/s1, Figure S1: Chromatograms of SCFAs and their internal standards (IS) of a representative fecal sample—females; Figure S2: Chromatograms of SCFAs and their internal standards (IS) of a representative fecal sample—males; Figure S3: Scatter plots of statistically significant correlations of anthropometric, biochemical, and lifestyle parameters and fecal SCFAs—females; Figure S4: Scatter plots of statistically significant correlations of anthropometric, biochemical, and lifestyle parameters and fecal SCFAs—males.
