# Supplementary material for: The Potential Role of SCFAs in Modulating Cardiometabolic Risk by Interacting with Adiposity Parameters and Diet
Source: Nutrients. 2024 Jan 16;16(2):266. doi: 10.3390/nu16020266 (PMC10818948; doi:10.3390/nu16020266)
Supplement: Supplementary file 1 [file nutrients-16-00266-s001.zip › nutrients-2801344-supplementary.pdf]

nutrients MDPI

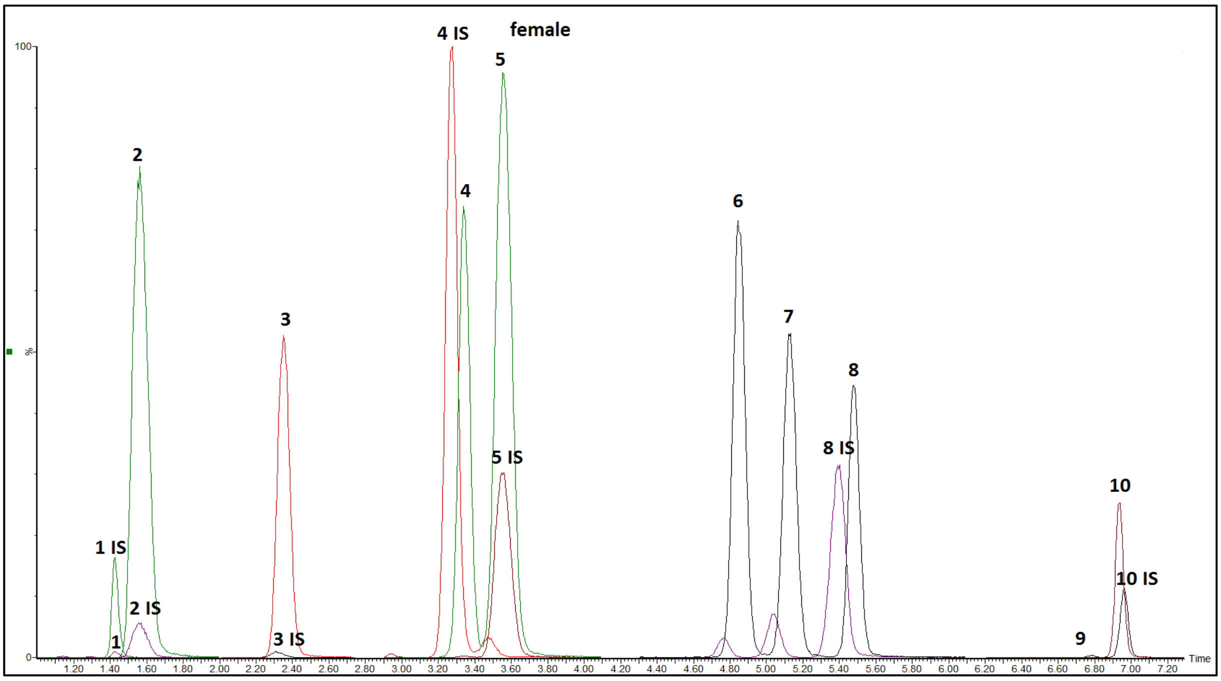

Supplementary Figure S1. Chromatograms of SCFAs and their internal standards (IS) of a representative fecal sample – females. **2** - C 2:0—acetic acid (AA), **3**- C 3:0—propionic acid (PA), **4** - C 4:0 i— isobutyric acid (IBA), **5** - C 4:0 n butyric acid (BA), **7** - C 5:0 i— isovaleric acid (IVA), **8** - C 5:0 n—valeric acid (VA), **9** - C 6:0 i— isocaproic acid (ICA), **10** - C 6:0 n—caproic acid (CA). **1** and **6** – data not analyzed in this study.

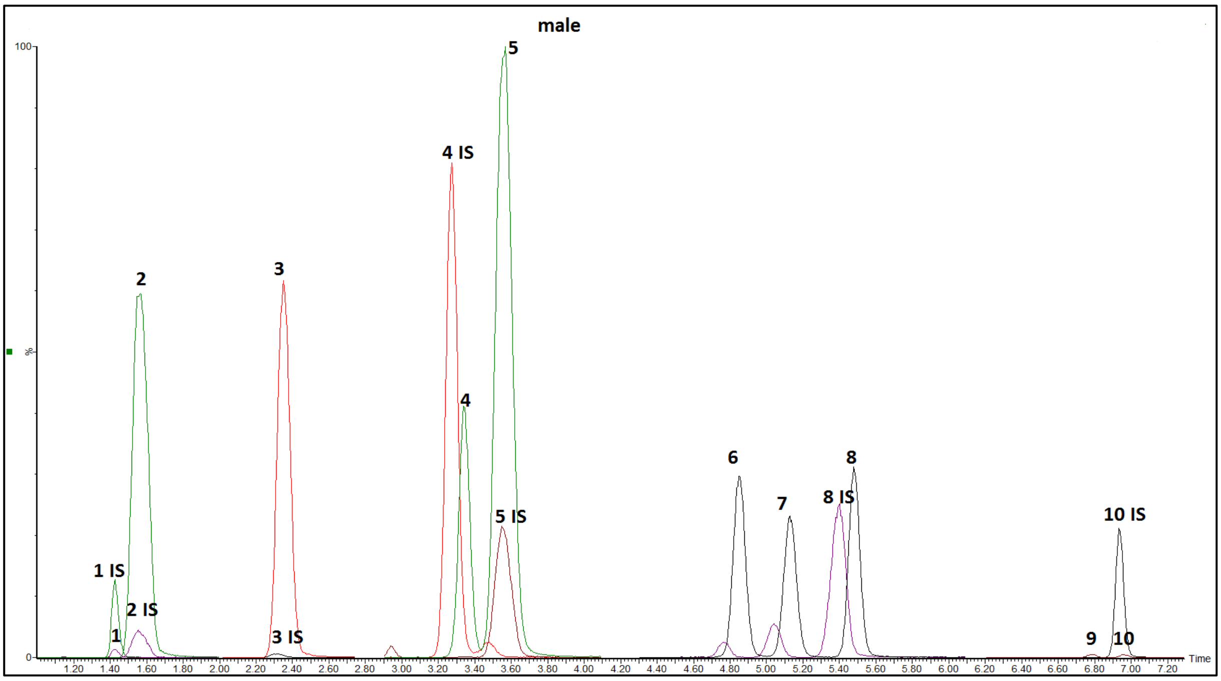

Supplementary Figure S2. Chromatograms of SCFAs and their internal standards (IS) of a representative fecal sample – males . **2** - C 2:0—acetic acid (AA), **3**- C 3:0—propionic acid (PA), **4** - C 4:0 i— isobutyric acid (IBA), **5** - C 4:0 n butyric acid (BA), **7** - C 5:0 i— isovaleric acid (IVA), **8** - C 5:0 n—valeric acid (VA), **9** - C 6:0 i— isocaproic acid (ICA), **10** - C 6:0 n—caproic acid (CA). **1** and **6** – data not analyzed in this study.

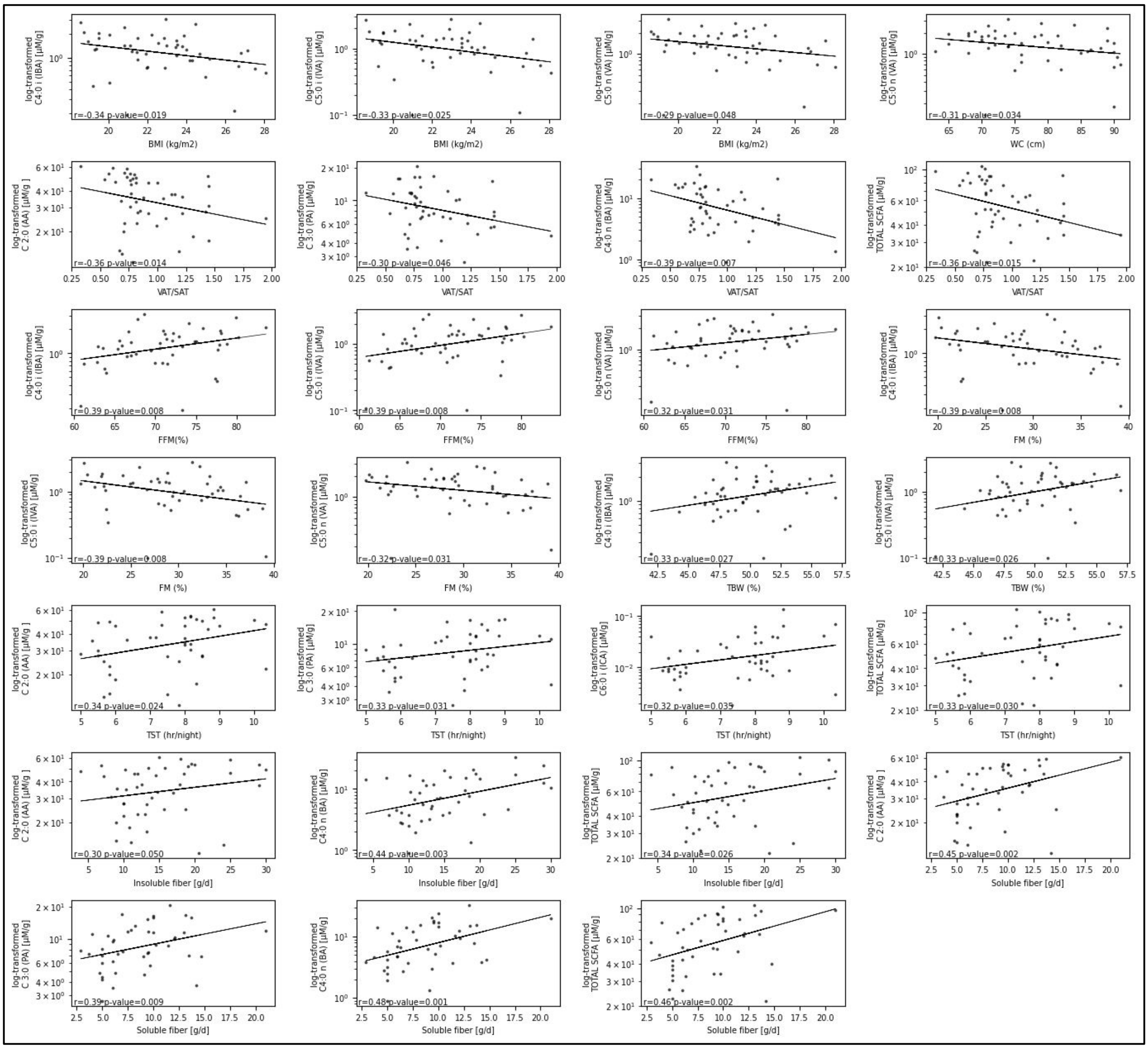

Supplementary Figure S3. Scatter plots of statistically significant correlations of anthropometric, biochemical, and lifestyle parameters and fecal SCFAs – females.

nutrients

MDPI

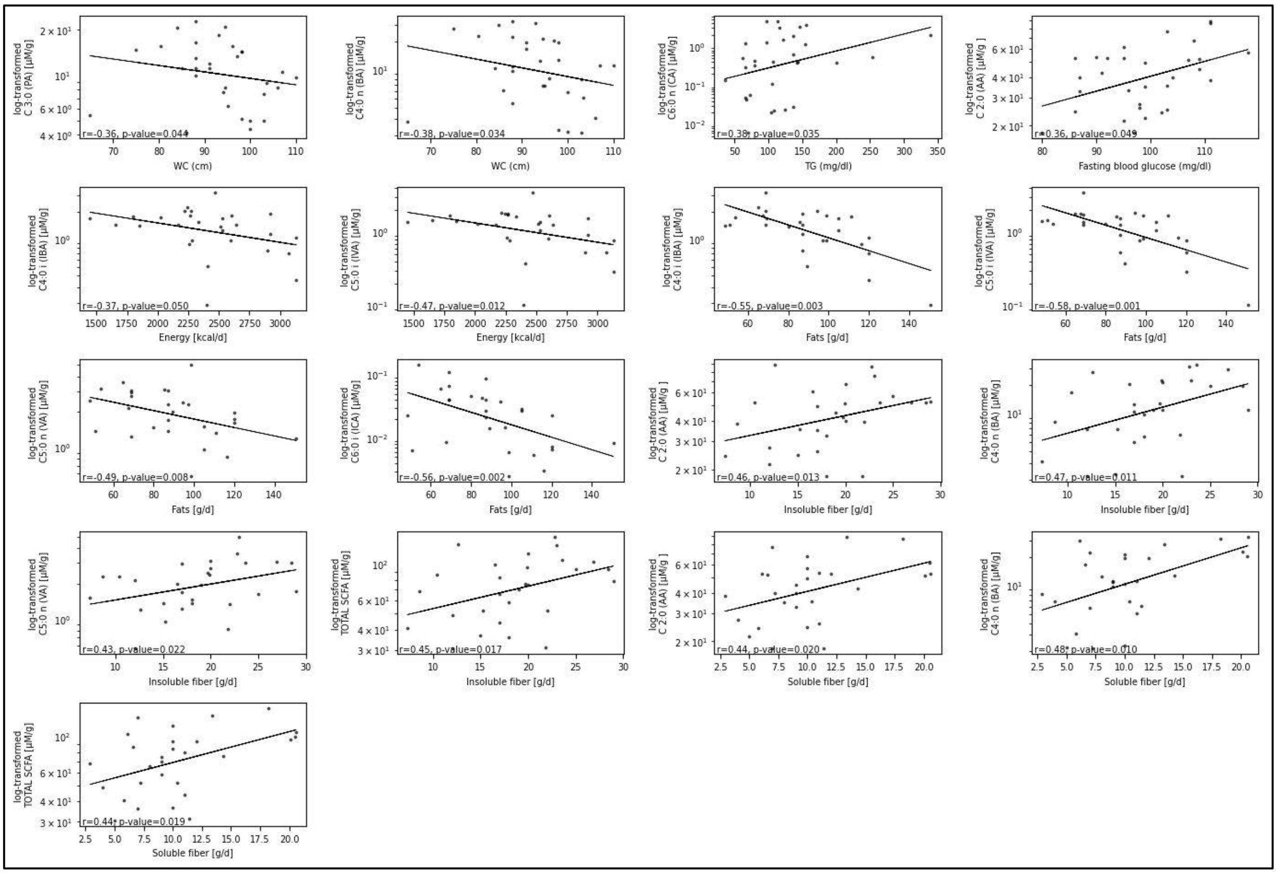

Supplementary Figure S4. Scatter plots of statistically significant correlations of anthropometric, biochemical, and lifestyle parameters and fecal SCFAs – males
